# A Regimen of Taxol, Ifosfamide, and Platinum for Recurrent Advanced Squamous Cell Cancer of the Anal Canal

**DOI:** 10.1155/2011/163736

**Published:** 2011-06-15

**Authors:** Diana V. Golub, A. Cahid Civelek, Vivek R. Sharma

**Affiliations:** ^1^The Ohio State University Medical Center, 395 West 12th Avenue, 3rd Floor, Columbus, OH 43210, USA; ^2^Department of Radiology, University of Louisville, 530 South Jackson Street, Suite C07, Louisville, KY 40202, USA; ^3^University of Louisville, James Graham Brown Cancer Center, 529 South Jackson Street, Louisville, KY 40202, USA

## Abstract

The typically recommended chemotherapy options in metastatic anal cancer generally yield partial remissions with limited benefit for the majority of patients. TIP is a regimen containing paclitaxel (Taxol), ifosfamide, and cisplatin that is known to have significant activity in patients with squamous cell cancers of the head and neck as well as in cervical cancer, both of which are malignancies strongly associated with oncogenic strains of human papilloma virus (HPV). Interestingly, squamous cell cancer of the anal canal shares an almost identical pathophysiology including causal association with HPV. Due to this, we chose to use the TIP regimen to treat patients with advanced anal cancer at our institution and report our findings on three such consecutive patients. All the patients tolerated the regimen well with manageable side effects and had excellent responses with complete resolution of PET activity after treatment. Our observations suggest that TIP is highly active for squamous cell cancer of the anal canal and warrants further study in the treatment of this disease.

## 1. Introduction

While representing only two percent of digestive tract cancer [1], the number of anal squamous cell cancer cases has continued to increase over the last few decades [2]. This phenomenon has been linked to numerous factors, the most significant of which is infection with human papilloma virus (HPV) [3]. In the past, this malignancy was treated primarily by inguinal node dissection and abdominoperineal resection (APR), resulting in the need for a colostomy. Five-year survival rates ranged from forty to seventy percent [4, 5]. Today, a regimen involving concurrent radiation and chemotherapy, the latter utilizing 5-fluorouracil and mitomycin-C, is the standard of care with surgery being reserved for salvage in case of residual disease or local recurrence after chemoradiation (CRT). This sphincter-preserving approach has resulted in the majority of patients being cured with CRT alone thereby avoiding a permanent colostomy [6, 7].

While this organ-preserving strategy has improved the overall quality of care and long-term outcomes of patients with localized disease, options for patients with more advanced or metastatic anal cancer remain relatively limited and primarily involve combination chemotherapy. A regimen consisting of 5-fluorouracil, and cisplatin has been the most frequently studied and results in overall response rates of around sixty percent, most of which are partial responses [8]. The median survival is approximately twelve months. A more aggressive combination of mitomycin-C, doxorubicin and cisplatin followed by bleomycin and CCNU yielded relatively similar outcomes [9]. Smaller reports have included carboplatin, doxorubicin, and irinotecan with or without cetuximab. Again, partial remissions are the norm [10–14]. 

 We present our experience treating patients with recurrent, metastatic anal cancer using a regimen consisting of a combination of paclitaxel, ifosfamide, and cisplatin (TIP). We chose to use this regimen due to the impressive activity it has shown in squamous cell cancers of the head and neck [15] and the uterine cervix [16], both of which share a striking resemblance to anal cancer. Like anal cancer, they are both squamous cell malignancies whose pathogenesis has been strongly associated with oncogenic strains of HPV [17, 18]. Due to this etiopathologic homology, it was conceivable that anal cancer may respond just as well to this treatment, and we report the results of our observations here.

## 2. Methods and Results

The treatment protocol and subsequent response and survival of three consecutive patients at our institution with recurrent, advanced squamous cell cancer of the anal canal are the focus of this paper. All three patients were African American females in the forty-to fifty-year age range, with minimal comorbidities, initially treated with 5- fluorouracil and cisplatin concurrently with radiation therapy. Subsequently, following development of recurrent disease all patients got the same regimen consisting of paclitaxel 175 mg/m^2^ given on day 1, ifosfamide 1 g/m^2^ daily on days 1 to 4 and cisplatin 75 mg/m^2^ on day 1. Cycles were repeated at 3 week intervals and appropriate supportive measures including dose adjustments or delays were instituted based on previously reported experience with the regimen in other malignancies [15, 16]. All patients received growth factor prophylaxis to prevent febrile neutropenia.

**Case 1**

**Figure 1:**
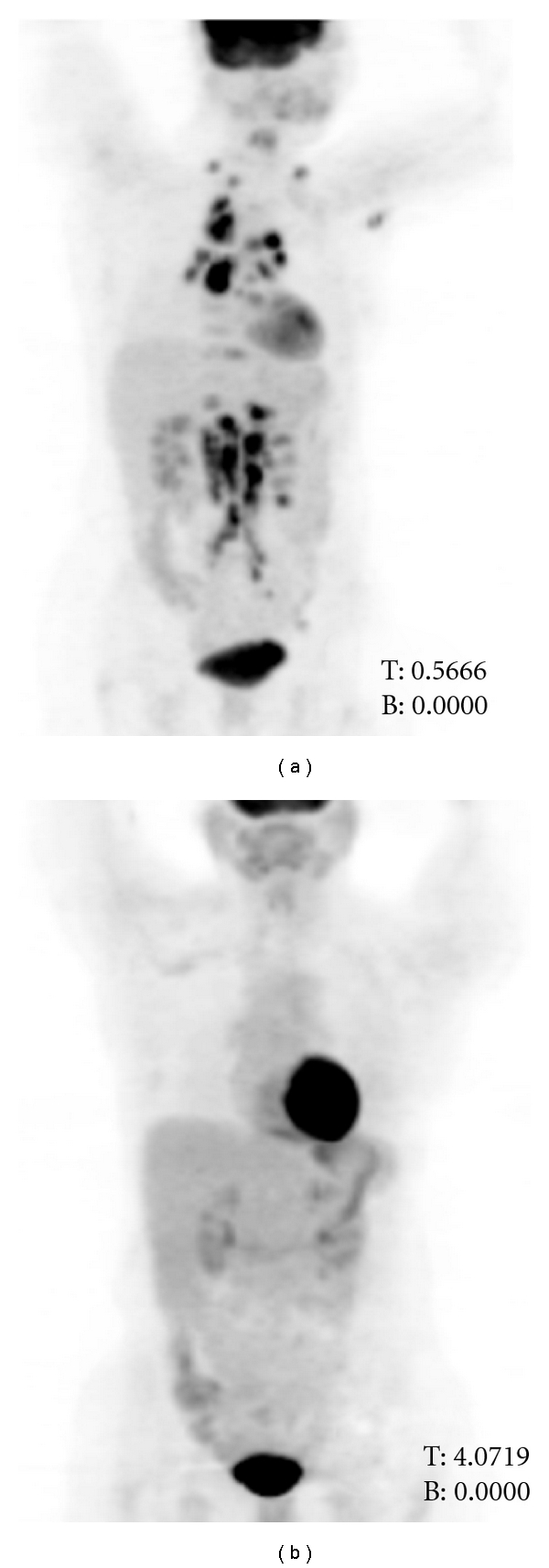
(a) The pretherapy anterior (0° degree) maximum intensity projection image of the PET/CT study of patient 1 showing multiple metastatic lesions in bilateral supraclavicular fossa, throughout the mediastinum, hila and in abdominal nodal basins. (b) The six-months posttherapy anterior (0° degree) maximum intensity projection image of the PET/CT study of the patient showing complete resolution of the previously seen FDG avid lesions.

A 46-year-old female presented with complaints of difficulty with bowel movements and anal discomfort and was found to have an infiltrating poorly differentiated squamous cell carcinoma on anal mass tissue biopsy. Fine needle aspiration (FNA) of her bilateral inguinal lymph nodes was positive for metastatic squamous cell carcinoma and she was staged as T3N3M0 based on PET/CT imaging. Treatment with 5FU and cisplatin along with RT resulted in complete remission which was confirmed by tissue biopsy. Approximately nine months after completion of her treatment the patient presented with complaints of night sweats, back pain and had an enlarged supraclavicular lymph node on the right side of her neck. Imaging with PET/CT showed very extensive disease that appeared to primarily involve nodal areas (Figure 1(a)). Of note, there were no lesions seen in the inguinal lymph nodes or the anal region. Also, there was no evidence of a head and neck or lung primary by clinical examination or imaging. The patient underwent an FNA of the enlarged cervical lymph node which showed keratinizing squamous cell carcinoma consistent with anal primary. She was started on TIP chemotherapy every twenty one days with growth factor support. CT (without PET) done after three cycles showed complete resolution of all radiographic lesions and a subsequent PET/CT after three more cycles also showed complete resolution of FDG uptake (Figure 1(b)). The patient became asymptomatic and was able to discontinue all analgesics. She completed a total of six cycles of TIP chemotherapy and remained disease-free on serial imaging for six months after the first documentation of complete remission (CR). She subsequently recurred with widespread involvement of lymph nodes in the neck, mediastinum, right hilum, retroperitoneum, and pelvis and succumbed to the disease six months later.

**Case 2**

**Figure 2:**
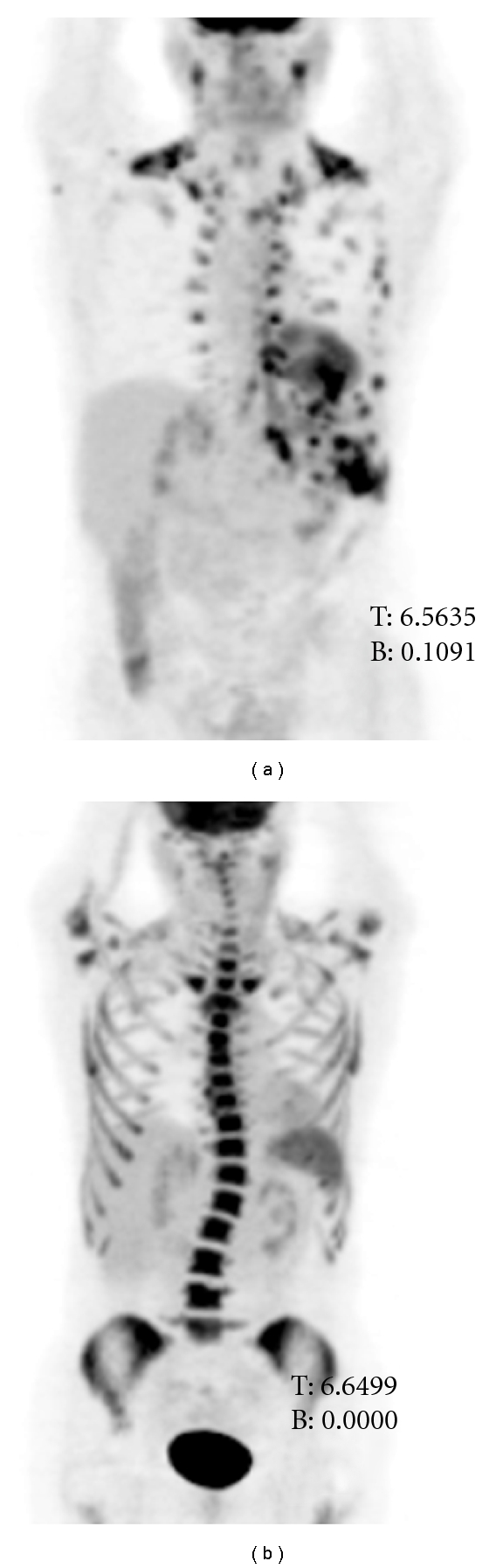
(a) The pretherapy anterior (0° degree) maximum intensity projection image of the PET/CT study of the patient demonstrating multiple pleural-based nodular metastatic lesions in the left chest along with prominent FDG uptake also seen in the activated brown fat tissue in multiple locations. (b) The six-months posttherapy anterior (0° degree) maximum intensity projection image of the PET/CT study of the patient showing complete resolution of the previously seen FDG avid lesions associated with intense bone marrow FDG activity and increased splenic activity seen due to therapy-induced bone marrow stimulation.

A 42-year-old female with four months of perirectal pain and intermittent rectal bleeding was found on examination to have an anal mass along with palpable inguinal lymph nodes bilaterally. Biopsies of the primary lesion as well as the inguinal lymph nodes revealed moderately differentiated keratinizing squamous cell carcinoma. A subsequent whole body PET/CT indicated a 5 cm anal mass, a 2.3 cm enlarged left iliac chain lymph nodes, enlarged left inguinal lymph nodes, and slightly prominent right inguinal nodes. Based on the above information, the patient was staged as T3N3M0 (IIIB) and treated with a 5FU/cisplatin regimen with concurrent radiation. She tolerated this well and remained disease-free for over a year at which time she developed persistent left thoracic and back pain. A chest CT indicated prominent pleural-based densities and pleural thickening in the left hemithorax with a PET/CT scan showing an area of intense hypermetabolism in the left pleural compartment (Figure 2(a)). A CT-guided biopsy of the pleural-based mass was done, which confirmed the diagnosis of recurrent squamous cell anal carcinoma after careful comparison of the histology with that of the original primary tumor. At this point, the TIP regimen was administered every twenty-one days, along with growth factor support. The patient tolerated treatment fairly uneventfully and only had mild (grade 1 or 2) hematologic and GI toxicities (Table 1). After the first three cycles, a follow-up PET/CT scan indicated significant shrinkage of multifocal lobular-nodular based metastases in the lung with complete resolution of FDG uptake (Figure 2(b)). She went on to get a total of six cycles and remained disease-free for two and a half years. At her most recent appointment, patient had pleuritic chest pain and was found to have a mass lining the left hemidiaphragm. A biopsy was attempted but was unsuccessful due to the difficult location of the tumor and patient has begun treatment with TIP with the presumptive diagnosis of recurrent anal cancer.

**Case 3**

**Figure 3:**
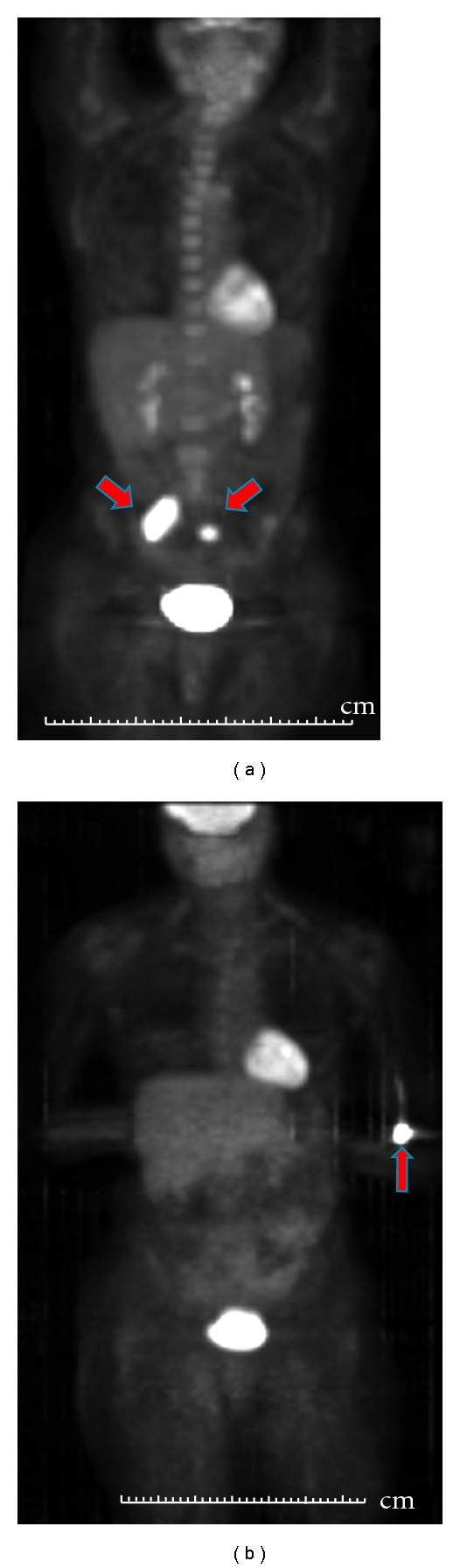
(a) The pretherapy anterior (0° degree) maximum intensity projection image of the PET/CT study of the patient is shown with the red arrows indicating the right internal iliac and left common iliac lesions. (b) The four-months posttherapy anterior (0° degree) maximum intensity projection image of the PET/CT study shows resolution of previously seen FDG avid bilateral iliac lesions while the arrow indicates the FDG activity at left antecubital fossa injection site.

A 54-year-old female with rectal bleeding and weight loss was found to have a large anal mass along with left inguinal adenopathy. A biopsy of her anal mass and left groin indicated moderately differentiated squamous cell carcinoma and she was staged as T4N2M0. A diverting colostomy was done and the patient was treated with 5FU and cisplatin with concurrent radiation. Although she had complete resolution of her primary and inguinal disease, follow-up CT imaging showed development of a soft tissue mass in the pelvis deep to the right psoas muscle, also involving the iliac arteries, with progression of the disease indicated on further imaging (Figure 3(a)). At this point she was referred to our institution for further treatment options. We started her on the TIP regimen which she tolerated fairly well with only grade 1 or 2 hematologic toxicity (Table 1). A follow-up PET/CT scan after the second cycle of chemotherapy, revealed an interval decrease in the size of the right retroperitoneal mass and a substantial reduction in the FDG uptake. After two additional cycles, there was further reduction in tumor size and complete resolution of FDG uptake per PET/CT (Figure 3(b)). A PET/CT scan done after two additional cycles revealed stable findings of persistent soft tissue density between the L5 vertebral body and the right psoas muscle with no FDG uptake. The patient had completed 6 cycles of TIP but was not felt to be resectable due to vascular involvement and was therefore followed off therapy with another PET/CT in two months. This unfortunately showed disease progression with an increase in the size of the mass as well as FDG uptake and she became symptomatic again with pelvic and hip pain. She was treated with another cycle of TIP with symptomatic improvement but was unable to continue treatment due to socioeconomic and other personal reasons. She has since received palliative RT to the psoas mass with symptomatic relief and is alive with disease at the time of this writing.

## 3. Discussion

Squamous cell cancer of the anal canal comprises about two percent of the malignancies of the GI tract [1] and is now well known to be strongly associated with infection by oncogenic strains of HPV [3]. Clinically, it is associated with substantial morbidity and suffering including rectal bleeding and pain which is often severe. In the past, the mainstay of treatment was an APR which left patients with lifelong colostomies and all the accompanying quality-of-life issues. However, with the advent of an organ-sparing approach consisting of concomitant radiation and chemotherapy, the latter including mitomycin-C and cisplatin, approximately eighty percent of patients can be cured without the need for morbid surgery [19, 20]. The treatment regimen incorporates the use of 5-FU (1000 mg/m^2^ per day by continuous infusion days 1 through 4 and 29 through 32), mitomycin (10 to 15 mg/m^2^ on day 1 only), and an intermediate dose of radiation therapy (30 Gy). Due to the substantially higher hematologic toxicities associated with mitomycin-C, its substitution by cisplatin has been investigated in recent studies with conflicting results. In the RTOG 98-11 trial, there was no significant difference in five-year disease-free survival (54 versus 60 percent for the cisplatin and mitomycin groups, respectively; *P* = .17) or overall survival (70 versus 75 percent; *P* = .10). However, colostomy rates (cumulative rate 19 versus 10 percent; *P* = .02) were significantly higher in the induction cisplatin/5-FU arm [21]. The ACT II study on the other hand showed no difference between mitomycin-C or cisplatin containing regimens in any of the above parameters including colostomy rates [22].

The above issues notwithstanding, the high cure rates achieved with CRT alone represent a substantial step forward in the quality of care from the days of universal APRs. The benefit of this advancement however has been limited to patients with localized disease. Those that have more advanced disease at diagnosis or that recur with unresectable disease still have disappointingly few options available to them. Given that the vast majority of patients with anal cancer are cured with CRT with or without salvage surgery, it has been difficult to get enough numbers of patients with recurrent or metastatic disease to do large clinical trials in this setting. In terms of systemic therapy, the combination of 5FU and cisplatin has been the most extensively studied and reported [8]. Response rates have been between fifty to sixty percent with, most of which are partial. Similar results have been noted in other studies using combinations such as mitomycin, doxorubicin and cisplatin followed by bleomycin and CCNU or single agents like carboplatin, irinotecan with or without cetuximab [9–14].

All of the above regimens mostly induce a partial response (PR) which is certainly helpful in controlling the disease for a period of time. They typically however, do not appear to induce complete remissions (CR). This also limits their usefulness in patients with advanced disease that is potentially amenable to local therapy. In this setting, therefore, it would be very helpful to have a regimen that induces a high-rate of response including a high proportion of CRs so that those who have a chance of getting potentially curative local therapy may be more likely to do so. Since the small numbers make it difficult to generate high quality data defining a standard in this situation, extrapolation from other diseases with biologic similarities to anal cancer would be a reasonable strategy to find more active treatment options for this subset of patients. It has been noted that squamous cell carcinoma of the anal canal is strikingly similar to squamous cell cervical cancer and squamous cell carcinoma of the head and neck both in terms of histology and a strong causative association with HPV infection [3, 17, 18]. Patients with these head and neck and cervical cancers have been shown to have impressive responses following the administration of TIP chemotherapy [15, 16]. In the study with recurrent head and neck cancer by Shin et al. [15] the overall response rate with TIP was 58% with 17% complete remissions. In the subset of patients with metastatic disease, the response rate was even higher at 80%. The toxicity was primarily hematologic with a 27% incidence of febrile neutropenia albeit in the absence of growth factor prophylaxis. The only other significant toxicity besides alopecia and nausea was peripheral neuropathy which was grade 3 in 5% of the patients. Similar results were reported by Zanetta et al. with the use of TIP for recurrent cervical cancer [16]. Given the pathobiologic similarity of these malignancies to squamous cell cancer of the anal canal, it is conceivable that similar high quality responses may be attainable in this disease as well. Our experience with the three patients presented here certainly point to significant activity of TIP in anal cancer. All the patients had a rapid and dramatic response with complete remission (CR) per PET scanning in all three cases and a CR by RESIST criteria in two of the three. Especially interesting is the second patient who remained disease-free more than two years after completion of TIP chemotherapy for her biopsy proven metastatic recurrence. It must be emphasized that the TIP regimen typically requires inpatient administration and carries significant risk of hematologic toxicity. In the study by Shin et al. [15] even though anemia and thrombocytopenia were mostly mild or moderate, the incidence of febrile neutropenia was 27% in the absence of growth factor prophylaxis. It is therefore imperative that patients be carefully selected for performance status and tolerance and closely monitored for supportive care during treatment as per lessons gleaned from the use of this regimen in other cancers. We chose to use prophylactic growth factor support to prevent febrile neutropenia and none of our patients had this toxicity. As long as these guidelines are followed, patients appear to tolerate the regimen remarkably well and only one patient in our series had a grade 3 adverse event which was peripheral neuropathy (Table 1). 

 In conclusion, this is the first report of the use of TIP chemotherapy in anal cancer. We believe our observations suggest that this is a highly active regimen in this setting and may be a promising avenue of future study not only for patients with recurrent or metastatic disease but also for treatment of selected high-risk patients with localized and potentially curable disease.

## Figures and Tables

**Table 1:** Summary of patient characteristics and treatment outcomes with TIP.

Patient	Stage at original diagnosis	Treatment at original diagnosis	Presentation at recurrence	Treatment at recurrence	Toxicity besides alopecia with TIP: all were NCI grade 1 or 2 unless otherwise specified	Best response to TIP by RECIST criteria	Duration of response with TIP	Survival since recurrence
Case 1-46-year-old female	T3N3M0	CRT with 5FU/cisplatin	Biopsy-proven metastatic disease in chest and abdomen twelve months after original diagnosis	TIPx6 cycles then TIPx2 cycles then FOLFOX6 modified x1 cycle	Anemia Thrombocytopenia PN (grade 3)	CR per CT after 3 cycles confirmed by PET/CT after 3 more cycles	6 months	14 months

Case 2-42-year-old female	T3N3M0	CRT with 5FU/cisplatin	Biopsy-proven metastatic disease in chest twenty two months after original diagnosis	TIPx6 cycles	Anemia Thrombocytopenia Diarrhea Syncope PN	CR per PET/CT after 3 cycles	2 years and six months	2 years and six months thus far

Case 3-54-year-old female	T4N2M0	CRT with 5FU/cisplatin	Metastatic disease in pelvis five months after original diagnosis	TIPx7 cycles then RT to right paraspinal mass	Anemia Thrombocytopenia Nausea	CR per PET after 4 cycles (PR per CT)	4 months	17 months thus far (patient alive with disease)

CRT: chemo radio therapy

5FU: fluorouracil

CR: complete remission

PR: partial remission

RECIST: response evaluation criteria in solid tumors

FOLFOX: infusional fluorouracil + leucovorin + oxaliplatin.

PN: Peripheral neuropathy.
